# New insights into the management of homozygous familial hypercholesterolemia patients treated with lomitapide: a single-center experience

**DOI:** 10.3389/fendo.2024.1515846

**Published:** 2024-12-24

**Authors:** Gabriella Iannuzzo, Ilenia Lorenza Calcaterra, Marco Gentile, Claudia Stanzione, Francesca de Ruberto, Maria Donata di Taranto, Giovanna Cardiero, Giuliana Fortunato, Matteo Di Minno

**Affiliations:** ^1^ Department of Clinical Medicine and Surgery, University of Naples Federico II, Naples, Naples, Italy; ^2^ Department of Molecular Medicine and Medical Biotechnologies, CEINGE Advanced Biotechnologies S.C. A.R.L, University of Naples Federico II, Naples, Italy

**Keywords:** HoFH, hypercholesterolemia, lomitapide, management, monitoring

## Abstract

Familial hypercholesterolemia (FH) is a genetic disease, usually with onset during childhood, characterized by elevated blood LDL cholesterol levels and potentially associated with severe cardiovascular complications. Concerning mutated genes in FH, such as *LDLR*, a small subset of FH patients presents a homozygous genotype, resulting in homozygous FH (HoFH) disease with a generally aggressive phenotype. Besides statins, ezetimibe and PCSK9 inhibitors, lomitapide (an anti-ApoB therapy) was also approved in 2012–2013 as an adjunctive treatment for HoFH. Despite its clinical efficacy, lomitapide administration should be done with caution because of the possible occurrence of side effects, such as hepatosteatosis, increased blood transaminase levels and gastrointestinal symptoms, as well as the possible deleterious interactions with other drugs. In this context, we decided to report the main available evidence on the management and monitoring of HoFH patients treated with lomitapide and to accompany this literature review with a description of our clinical experience with a subset of six HoFH patients. In conclusion, this paper aims to address an important topic for HoFH-related clinical practice that, to our knowledge, is not yet formally regulated by proper national and/or international guidelines.

## 1 Introduction

Familial hypercholesterolemia (FH) is a genetic disease that is distinguished by abnormally high levels of low-density lipoprotein (LDL) cholesterol, detectable even in early childhood, and can lead to severe cardiovascular (CV) complications (1). A particular and rare form of this disease is the homozygous FH (HoFH), which features a homozygous genotype, thus harboring both alleles with the same mutation. The genetic lesions of HoFH usually involve one of the following genes: *LDLR* (90% of HoFH patients carry biallelic mutations of this gene), *APOB, PCSK9* and *LDLRAP1* (2). Besides the homozygous genotype, there can also be compound and double heterozygosity cases, namely different mutations in each allele and mutations in two alleles of two different genes, respectively. From a clinical point of view, these less frequent genotypes are linked to a more severe phenotype than that of the more common heterozygous FH (HeFH) (1).

From an epidemiologic point of view, the prevalence of HoFH varies between 1 in 300,000 and 1 in 360,000 people. However, these data can increase in geographically isolated populations and those populations with evidence of founder effect (2). Furthermore, considering a worldwide study that included 751 patients from 38 different countries from the HoFH International Clinical Collaborators registry, the median age at diagnosis was 12 years old, the distribution of patients by sex was fairly balanced (52% female and 48% male), and based on geographic origin, 64% of patients were white, 23% were Asian and 13% were black or mixed race (3).

The diagnosis of HoFH can be made clinically or genetically. In the first case, the clinical manifestations are LDL cholesterol levels >13 mmol/L (500 mg/dL) and/or the presence of xanthomas before the age of 10 years or having both parents with HeFH. In the second case, genetic alterations must be found in at least one of the four previously cited genes responsible for HoFH (3).

Given the severe CV complications that HoFH patients may develop, early diagnosis and timely initiation of lipid-lowering therapy (LLT) are crucial (2). First-line treatment for HoFH patients with statins (HMG-CoA reductase inhibitors) should be attempted but has limited efficacy, and add-on ezetimibe (the only available drug capable of inhibiting cholesterol absorption from the intestine) may be combined (1, 2, 4). Subsequently, depending on cost, availability, and patient’s residual LDLR activity, novel therapies may be added to the therapeutic regimen, particularly PCSK9 inhibitors (they increase LDL receptor expression on the cell membrane and thus enhance LDL cholesterol clearance), evinacumab (an anti-ANGPTL3 monoclonal antibody capable of reducing both LDL cholesterol and triglycerides [TGs]), and lomitapide (an anti-ApoB therapy). In addition to these drugs, plasma lipid apheresis can also effectively help control disease progression, having the ability to remove about 60% of LDL cholesterol from the blood. To prevent CV complications, this procedure should be started before the age of 6–7 years; despite its effectiveness, it does not always lead to successful clinical results because patients undergo this treatment irregularly, instead of the ideal weekly cadence, due to its invasive and time-consuming nature (2, 4).

As previously cited, lomitapide is among the novel drugs used for HoFH treatment. From a pharmacodynamic point of view, lomitapide is an inhibitor of microsomal TG-transfer protein, a cellular protein implicated in the production of ApoB-containing TG-rich lipoproteins in the liver and intestine. Therefore, lomitapide has an LDL-receptor-independent mechanism of action (2). Lomitapide was approved for clinical use by the Food and Drug Administration (FDA) and European Medicines Agency (EMA) in 2012 and 2013, respectively, as an adjunctive treatment to the standard LLT. The main study that proved the efficacy and safety of lomitapide was a single-arm, dose-escalation, open-label, phase III trial (ClinicalTrials.gov ID NCT00730236). Specifically, in a sample of 29 HoFH patients from the USA, Canada, South Africa and Italy, lomitapide was shown to induce a significant reduction in LDL cholesterol level from baseline of -50% at 26 weeks, -44% at 56 weeks and -38% at 78 weeks after starting the treatment. The safety profile was good overall, with some gastrointestinal (GI) symptoms as the most common side effects (5). Additionally, lomitapide has been shown to be safe and effective in the pediatric population in a case series (6), and the active clinical trial NCT04681170 is currently testing lomitapide in pediatric patients (2). Regarding the dosage, lomitapide should be taken orally with a starting dose of 5 mg once daily, moving to 10 mg after 2 weeks; then, lomitapide can be increased at 4-week intervals to 20 and 40 mg, up to a maximum dose of 60 mg daily, depending on the safety and tolerability profile (7).

**Figure 1:**
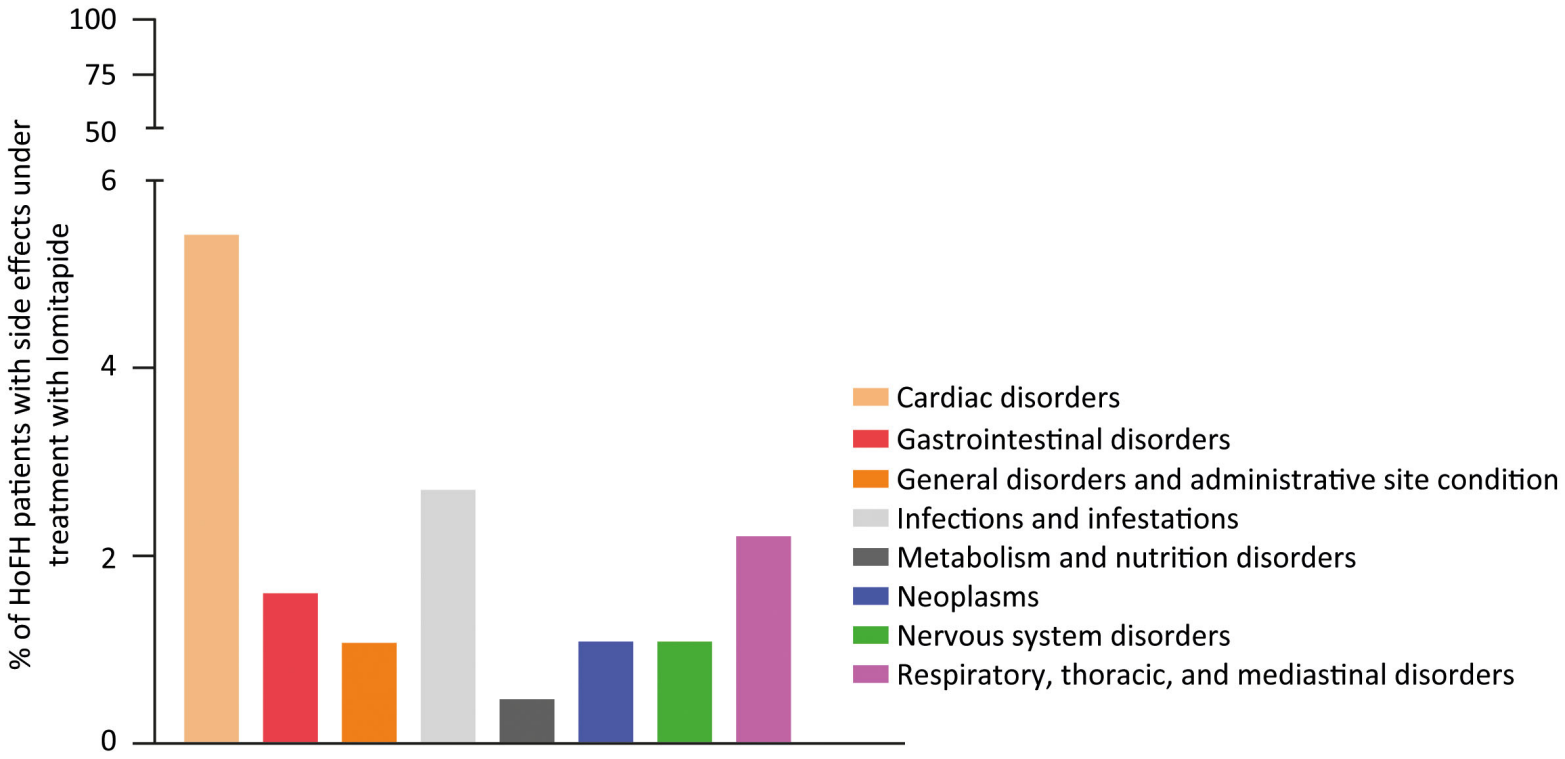
Trend of serious adverse events in HoFH patients treated with lomitapide. The follow-up period is 5 years, n=185. This graph was generated with GraphPadPrism software using from the data of the LOWER study (9).

Regarding the safety profile of lomitapide, the most common complications related to its intake are hepatosteatosis, increased blood transaminase levels and GI symptoms, such as nausea and diarrhea (2). The accumulation of fat inside the liver (hepatosteatosis) may occur in a mild-to-moderate manner without translating into a major clinical manifestation; indeed, biomarkers of hepatic damage and stiffness remain normal (8). To manage the elevation of hepatic enzymes, reducing or temporarily stopping the lomitapide dose appeared to be a viable method and allowed the continuation of therapy, as shown in the LOWER study (9). Finally, GI symptoms have been shown to become less severe and have a lower incidence throughout continued lomitapide treatment (2). A complete list of serious adverse events recorded during a 5-year follow-up period from the LOWER study is summarized in **Figure 1**.

To our knowledge, comprehensive and clear guidelines for monitoring and managing complications that may occur in HoFH patients treated with lomitapide are still lacking in the scientific literature. For this reason, we decided to gather all the main available evidence on this important topic, which still represents an unmet clinical need regarding HoFH, and to accompany this literature background by reporting our clinical experience on a relatively small subset of six HoFH patients.

## 2 Literature background

In this section of the manuscript, we aimed to gather all the main literature evidence regarding the monitoring and management of the adverse events that may occur in HoFH patients treated with LLT, also focusing on side effects related to lomitapide treatment. With this purpose, we performed a literature review on PubMed using the following search string: ((“homozygous familial hypercholesterolemia”[Title/Abstract]) OR (“homozygous familial hypercholesterolemia”[Title/Abstract]) OR (HoFH[Title/Abstract])) AND ((monitor*[Title/Abstract]) OR (complication*[Title/Abstract]) OR (management[Title/Abstract]) OR (“side effect”[Title/Abstract])) AND (lomitapide[Title/Abstract]). This search produced 55 results; by reading the title and abstract, 22 articles that matched the paper’s topic were selected. Following further and more thorough reading of the entire articles, 10 papers were finally selected as effectively related to the topic of this section of the present manuscript.

Knowing that CV complications are the most serious and likely adverse event that can occur in HoFH patients (1), careful monitoring of the CV apparatus’s health status is fundamental in these subjects. In this regard, the European Atherosclerosis Society has published consensus statements over the last decade to delineate optimal CV monitoring in HoFH patients. According to the 2014 guidelines, a general CV assessment should be performed in HoFH patients at diagnosis; next, an echocardiographic Doppler should be executed to assess the condition of the heart and aorta; then, during the follow-up period, patients should undergo computed tomography coronary angiography (CTCA) every 5 years. If CTCA cannot be accessed, stress testing or cardiac MRI may be valid alternatives. Overall, HoFH patients need to be followed by a multidisciplinary team of experts comprising a cardiologist to achieve therapy optimization and appropriate management of any CV complications (10). In 2023, the European Atherosclerosis Society updated the clinical guidelines for CV apparatus monitoring in HoFH patients. In particular, carotid plaque has been defined as an important marker of early-stage atherosclerosis, and its quantification should be performed using three-dimensional ultrasound; furthermore, MRI can help detect thrombosis and lipid enrichment in carotid plaques. Echocardiographic assessment of the heart and aorta should be performed annually after the diagnosis of HoFH. Moreover, HoFH patients presenting with symptoms of ischemia or valve malfunction should undergo invasive coronary angiography (11). Among CV complications, it is worth mentioning that pediatric HoFH patients may develop atheromatous CV disease; if this occurs, from a clinical management perspective, these children can be treated with aspirin with a dose range equal to 1–5 mg/kg/day. However, if fever and flu-like symptoms appear, aspirin should be discontinued to reduce the risk of Reye’s syndrome manifestation (12). As an example of the importance of cardiologic evaluation, Littmann et al. reported that a female HoFH patient receiving lomitapide treatment required referral to a cardiologist because of exertional dyspnea and bursts of chest pain; indeed, the echocardiogram revealed the presence of a tricuspid aortic valve, mildly increased scattered echogenicity, and mild to moderate central aortic insufficiency. 3 years later, in the same patient, CTCA detected the presence of a calcified plaque in the right coronary artery and the thoracic aorta (13). In light of the above and this clinical case experience, we can emphasize the importance of appropriate and routine CV imaging checks to monitor the CV status in HoFH patients.

Regarding GI symptoms linked to lomitapide administration, clinical practice has shown that a low-fat diet may be sufficient to successfully manage GI complications (14–17). In some cases, partial modification of the dietary regimen, lomitapide dose adjustment, or suspension may be necessary to make the GI manifestations disappear (14, 17). Another GI tract disorder related to lomitapide intake is elevated blood alanine aminotransferase (ALT) and aspartate aminotransferase (AST) hepatic enzyme levels. Therefore, the monitoring frequency of these biomarkers becomes important for the health of HoFH patients; indeed, hepatic enzymes should be measured before every lomitapide dose increase or monthly during the first year of treatment and at least every 3 months thereafter (18). Even in the case of liver injury with hepatic enzymes above the physiological levels, as with GI symptoms, lomitapide discontinuation or dose adjustment represents the main management approach to this health problem (13, 16–18). Finally, it should be mentioned that hepatosteatosis may be another side effect related to lomitapide intake. In this regard, careful monitoring of liver condition is crucial for the patient’s health; hence, liver biopsy, MRI and transient elastography can help detect and quantify hepatosteatosis and liver fibrosis (13).

As in the case of other pharmacological treatments, drug–drug interaction should be considered when administering lomitapide. *In vitro* studies showed that lomitapide is a direct inhibitor of CYP3A4, an enzyme deputed to metabolize several drugs within the human body. Statins are also metabolized by CYP3A4; therefore, high doses of lomitapide may suppress statin clearance by CYP3A4 and, as a result, expose the organism to serious side effects related to excessive statin exposure, such as myopathy and rhabdomyolysis. For this reason, careful attention must be paid to balance statin therapy with lomitapide treatment (19). Another example of drug–drug interactions comes from the clinical experience of the COVID-19 pandemic; some medications used for COVID-19, such as lopinavir, ritonavir, IFN-β1 and IFN-α and azithromycin, were revealed capable of increasing lomitapide exposure and, therefore, may amplify lomitapide-related side effects, such as hepatotoxicity. In light of this, lomitapide discontinuation should be done in HoFH patients who are also acutely ill with COVID-19 and treated with antimicrobial drugs for which significant drug–drug interaction has been shown (20). **Supplementary Table 1** summarizes the main known pharmacological interactions between lomitapide and other drugs, providing an essential overview for remembering the multiple drug–drug interactions and diversifying the timing of drug intake, especially given the mechanism of action of lomitapide. It should be noted that the table content was derived from the technical data sheets of single drugs and the work by Darpo and colleagues (21).

## 3 Patients

In this section, we reported the main clinical and biochemical features of a subset of six HoFH patients treated at the outpatient clinic of the Regional Reference Center for the Treatment of Dyslipidemia at AOU Federico II in Naples. These characteristics are summarized in **Table 1**.

**Table 1:** Clinical and biochemical features of a case series of six HoFH patients.

Features	Patient 1	Patient 2	Patient 3	Patient 4	Patient 5	Patient 6
Sex	Female	Female	Female	Male	Male	Male
Current age (years)	46	31	62	47	36	44
Age at diagnosis (years)	4	16	55	5	14	12
Diagnosis	Due to xanthomas and total cholesterol equal to 600 mg/dL	Due to xanthomas and total cholesterol equal to 500 mg/dL	Due to xanthomas and total cholesterol equal to 600 mg/dL	Genetic diagnosis at 5 years old and subsequent clinical diagnosis at 20 years old for xanthomas and corneal arches and total cholesterol equal to 720 (mg/dL)	Due to xanthomas and corneal arches and total cholesterol equal to 550 mg/dL	Due to tendon xanthoma and total cholesterol equal to 800 mg/dL
Therapy/age	□ Rosuvastatin from 40 to 46 years old □ Ezetimibe from 40 to 46 years old □ Lomitapide from 40 to 46 years old □ PCSK9i from 43 to 46 years old □ Lipid apheresis from 32 to 43 years old	□ Atorvastatin from 26 to 31 years old □ Ezetimibe from 26 to 31 years old □ Lomitapide from 27 to 28 years old □ PCSK9i from 28 to 31 years old □ Lipid apheresis never performed	□ Rosuvastatin from 55 to 60 years old □ Ezetimibe from 55 to 60 years old □ Lomitapide from 55 to 60 years old □ PCSK9i from 56 to 60 years old □ Lipid apheresis never performed	□ Atorvastatin from 43 to 47 years old □ Ezetimibe from 43 to 47 years old □ Lomitapide from 43 to 47 years old □ PCSK9i from 43 to 47 years old □ Lipid apheresis never performed	□ Statins from 14 to 36 years old □ Ezetimibe from 31 to 36 years old □ Lomitapide and PCSK9i: timing of use not available □ Lipid apheresis never performed	□ Rosuvastatin from 35 to 44 years old □ Ezetimibe from 35 to 44 years old □ Lomitapide from 41 to 44 years old □ PCSK9i from 40 to 44 years old □ Lipid apheresis from 32 to 43 years old □ PUFA from 35 to 44 years old
Familial history	Heterozygous parents and brother who died at 32 years from heart disease	Brother affected by HoFH	Children and parents with hypercholesterolemia	Data not available	Both parents with hypercholesterolemia	Parents who died at a young age from myocardial infarction; twin children with hypercholesterolemia
Genotype	Compound heterozygosis □ Mutation 1: [c.1646G>A], p.(G549D) □ Mutation 2: [c.1739C>T], p.(S580F)	Homozygosis □ Mutation 1: [c.1775G>A] p.(G592E) □ Mutation 2: [c.1775G>A], p.(G592E)	Compound heterozygosis □ Mutation 1: c.941-?_2311+?dup, p.(G314_Q770dup) □ Mutation 2: c.974G>A, p.(C325Y)	Homozygosis □ Mutation 1: [c.1135T>C], p.(C379R) □ Mutation 2: [c.1135T>C] [c.1135T>C], p.(C379R)	Homozygosis □ Mutation 1: [c.1775G>A], p.(G592E) □ Mutation 2: [c.1775G>A], p.(G592E)	Compound heterozygosis □ Mutation 1: [c.367T>C], p.(S123P) □ Mutation 2: [c.1478_1479delCT], p.(S493Cfs*42)
Types of therapy	□ Rosuvastatin (20 mg/day) □ Ezetimibe (10 mg/day) □ Lomitapide (5−40 mg/day) □ PCSK9i/evinacumab* (15 mg/day) □ PCSK9i/alirocumab (150 mg/day) □ Lipid apheresis (twice per week)	□ Atorvastatin (40 mg/day) □ Ezetimibe (10 mg/day) □ Lomitapide (5−10 mg/day) □ PCSK9i/evinacumab* (15 mg/day) □ PCSK9i/alirocumab (150 mg/day)	□ Rosuvastatin (20 mg/day) □ Ezetimibe (10 mg/day) □ Lomitapide (5−10 mg/day) □ PCSK9i/evolocumab (420 mg/day)	□ Atorvastatin (40 mg/day) □ Ezetimibe (10 mg/day) □ Lomitapide (20 mg/day) □ PCSK9i/evinacumab* (15 mg/day) □ PCSK9i/alirocumab (150 mg/day)	□ Atorvastatin (40 mg/day) □ Rosuvastatin (20 mg/day) □ Pravastatin (40 mg/day) □ Ezetimibe (10 mg/day) □ Lomitapide (5−20 mg/day) □ PCSK9i/alirocumab (150 mg/day) □ PCSK9i/evolocumab (420 mg/day)	□ Rosuvastatin (40 mg/day) □ Ezetimibe (10 mg/day) □ Lomitapide (5−10 mg/day) □ PCSK9i/evolocumab (140 mg/day) □ PUFA (2000 mg/day)
Cardiovascular events	CAD and PAD	Nothing	Systolic heart murmur	Unstable angina and aortic valve replacement	Nothing	Focal aortic and pulmonary murmur
Variation of LDL cholesterol during the period of follow-up (mg/dL)	249 → 42	411 → 39	514 → 158	620 → 120	587 → 166	690 → 30
Variation of total cholesterol during the period of follow-up (mg/dL)	346 → 94	500 → 89	600 → 205	720 → 163	550 → 233	800 → 68
Variation of HDL cholesterol during the period of follow-up (mg/dL)	36 → 42	39 → 35	46 → 30	32 → 32	34 → 33	45 → 26
Variation of triglycerides during the period of follow-up (mg/dL)	78 → 24	71 → 30	216 → 87	92 → 24	125 → 44	193 → 51
Variation of ALT enzyme levels during the period of follow-up (U/L)	13 → 21^†^	12 → 16^†^	52 → 53^†^	46 → 40	20 → 40^†^	30 → 51^†^
Variation of AST enzyme levels during the period of follow-up (U/L)	12 → 20^†^	20 → 14	40 → 51^†^	54 → 57^†^	17 → 23^†^	48 → 30
Variation of CPK enzyme levels during the period of follow-up (U/L)	142 → 87	98 → 126^†^	152 → 84	Data not available	136 → 218^†^	107 → 144^†^

*It is specified that evinacumab was administered in the context of a clinical trial through compassionate use (NCT0339978-6). ^†^Increase in hepatic enzymes. PCSK9i, Proprotein convertase subtilisin/kexin type 9 inhibitors; ALT, Alanine aminotransferase; AST, Aspartate aminotransferase; CPK, Creatinine phosphokinase; PUFA, Polyunsaturated fatty acid; CAD and PAD, Coronary or peripheral artery disease.The symbol → denotes "from - to."

Our case series consisted of three female and three male adult patients. The onset of HoFH occurred during childhood for all patients except for patient 3, who instead experienced HoFH for the first time in adulthood. The diagnosis always occurred because of the presence of xanthomas (lipid deposits) (1) and a total cholesterol (TC) level well above the cutoff to consider it elevated (240 mg/dL) (22). Regarding drug treatment, apart from patients 3 and 5, the other patients started pharmacological therapies several years after the initial diagnosis. This is mainly due to the fact that the first patients came to our Center at different times and ages, and some drugs, such as PCSK9 inhibitors and lomitapide, were approved by regulatory agencies only about 10 years ago (similarly, the pediatric use of statins obtained EMA and FDA approval only a few years ago) (23–25). Almost all patients were on standard LLT (statins + ezetimibe) plus novel drugs, such as lomitapide and PCSK9i, exactly as per the HoFH therapeutic algorithm (2, 4). Thus, considering both the familial history and the genotype for all patients, these two aspects together reflect the genetic character of HoFH.

The following section will discuss the hematochemical parameters of our six HoFH patients. First, in all patients, the TC level decreased significantly; in particular, for patients 1, 2, 4 and 6, it reached normal values (<200 mg/dL), and for patients 3 and 5, it was borderline (200–239 mg/dL) (22). These data underscore the overall good efficacy of drug therapy. As for LDL cholesterol levels, they significantly decreased during the follow-up period, although for patients 3, 4 and 5, the threshold level of <100 mg/dL for health safety was not reached (22). Although the pharmacological treatment was effective overall in our patients, it probably resulted in undesired effects. Besides light fluctuations, the high-density lipoprotein (HDL) cholesterol level was stable in all patients but with values <40 mg/dL, a risk factor for the development of CV diseases/complications (22), which were found in four out of six patients (patients 1, 3, 4 and 6). Specifically, CV complications were managed as follows: i) patient 1 underwent carotid thromboendoarterectomy; ii) patient 3 required revascularization and triple bypass; iii) patient 4 underwent a valve replacement surgery; and iv) patient 6 underwent revascularization. Similar to total and LDL cholesterol, TGs also showed a decrease in all patients, with good values (<150 mg/dL) (22). Regarding hepatic enzymes, ALT values increased in all patients (except for patient 4) but remained within the normal range (7–56 U/L) (26); increased AST was detectable in patients 1, 3, 4 and 5, while patients 3 and 4 had AST levels above the physiological range (10–40 U/L) (26). Finally, an increase in creatinine phosphokinase (CPK) levels was detected in patients 2, 5 and 6; however, only patient 5 exhibited a value out of the normal range (22–198 U/L) (26).

As previously reported, our patients experienced some adverse effects during drug therapy. Particularly, all six patients developed GI complications such as nausea and diarrhea, mainly linked to non-adherence to the diet; in this case, the symptoms improved through careful monitoring of the dietary regimen and, when necessary, with a short interruption of therapy (2–3 days). In the occurrence of hypertransaminasemia as a side effect, a pharmacological washout was carried out until transaminases normalized (on average 5–7 days); subsequently, therapy was reintroduced with a lower dosage, reserving the right to increase it, if necessary, also depending on the LDL cholesterol levels.

To manage side effects due to lomitapide administration, some patients of our case series (see **Table 1**) received evinacumab in combination with the other classical drugs and lomitapide (evinacumab was used in the context of a clinical trial through compassionate use [NCT0339978-6]). Specifically, we observed that adding evinacumab maintained HoFH disease control while decreasing the required dose of lomitapide and, therefore, reducing the likelihood of occurrence of potential side effects. This regimen has been successfully used by Tada et al. in a young boy with HoFH and resistant to statins and ezetimibe (27). In our clinical experience, this treatment regimen provided good prevention of adverse events. This is not yet a guideline for treating HoFH patients; however, to our knowledge, this is the first work showing such results with this therapeutic regimen. Importantly, if spread and used in other medical centers, this mode of managing patients with HoFH could find confirmation of its validity and actually become a standardized medical procedure.

**Figure 2:**
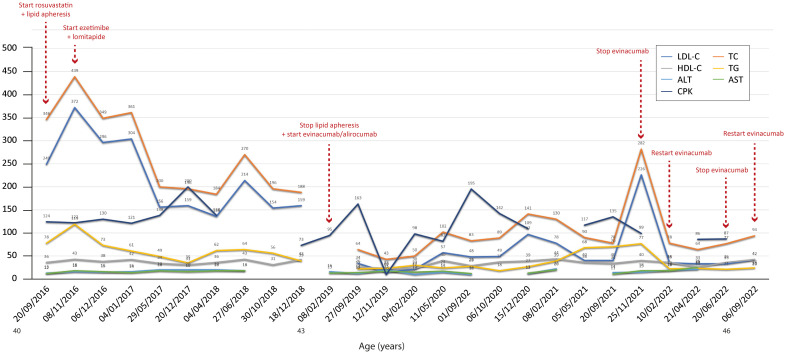
Temporal trends of cholesterol, hepatic enzymes and triglycerides in patient 1. The follow-up period was 6 years. Total and LDL cholesterol decreased overall during the period, but both increased when evinacumab was discontinued. The y-axis represents the blood levels of the various biochemical parameters examined. TC, LDL cholesterol, TGs and HDL cholesterol are measured in mg/dL, whereas ALT, AST and CPK are measured in U/L. TC, total cholesterol; LDL, low-density lipoprotein; HDL, high-density lipoprotein; TG, triglycerides; ALT, alanine aminotransferase; AST, aspartate aminotransferase; CPK, creatinine phosphokinase.

**Figure 3:**
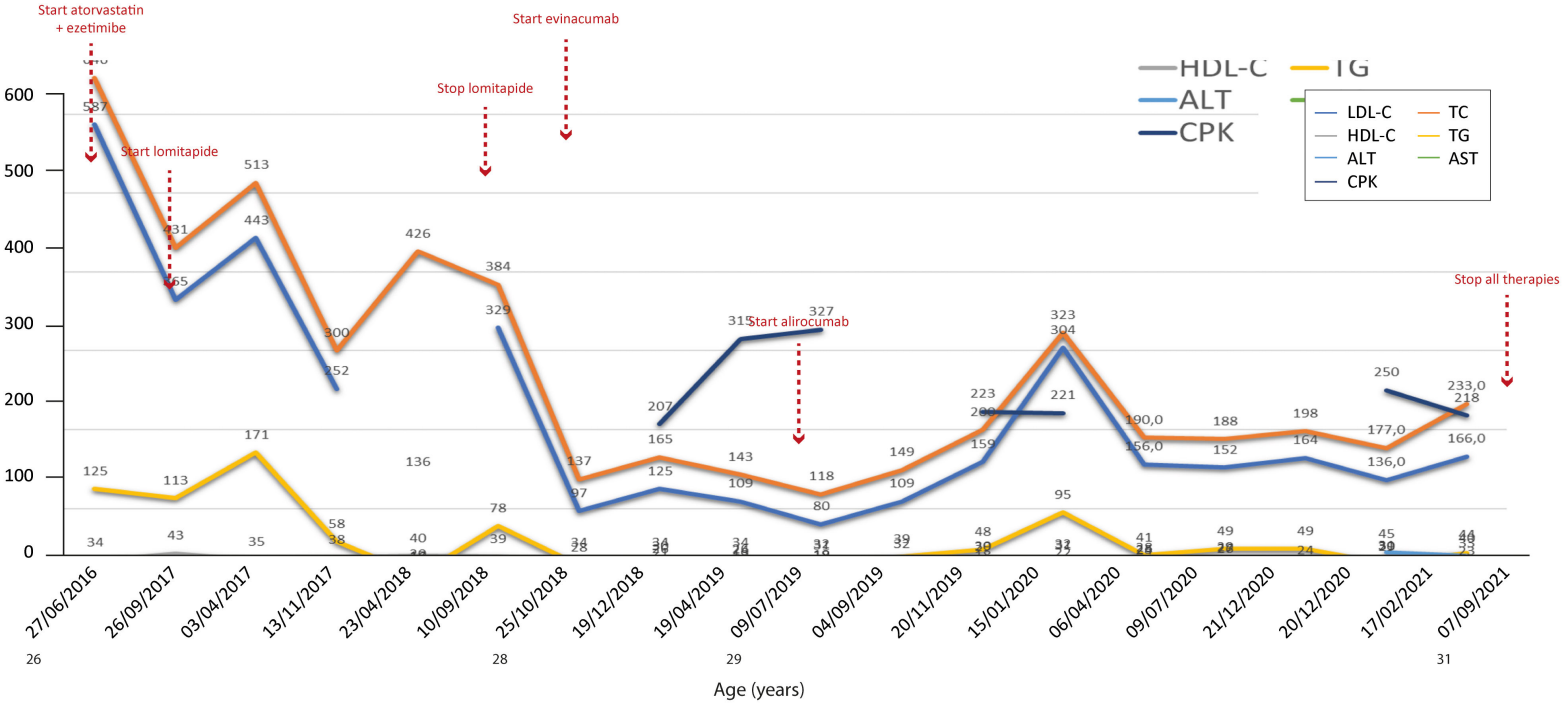
Temporal trends of cholesterol, hepatic enzymes and triglycerides in patient 2. The follow-up period was 5 years. Lomitapide was not as effective when considering that total cholesterol increased after starting the therapy; this could be due to an increase in HDL cholesterol, but the trend of the latter during the same period of increase in total cholesterol (2017–2018) is missing. The y-axis represents the blood levels of the various biochemical parameters examined. TC, LDL cholesterol, TGs and HDL cholesterol are measured in mg/dL, whereas ALT, AST and CPK are measured in U/L. TC, total cholesterol; LDL, low-density lipoprotein; HDL, high-density lipoprotein; TG, triglycerides; ALT, alanine aminotransferase; AST, aspartate aminotransferase; CPK, creatinine phosphokinase.

**Figure 4:**
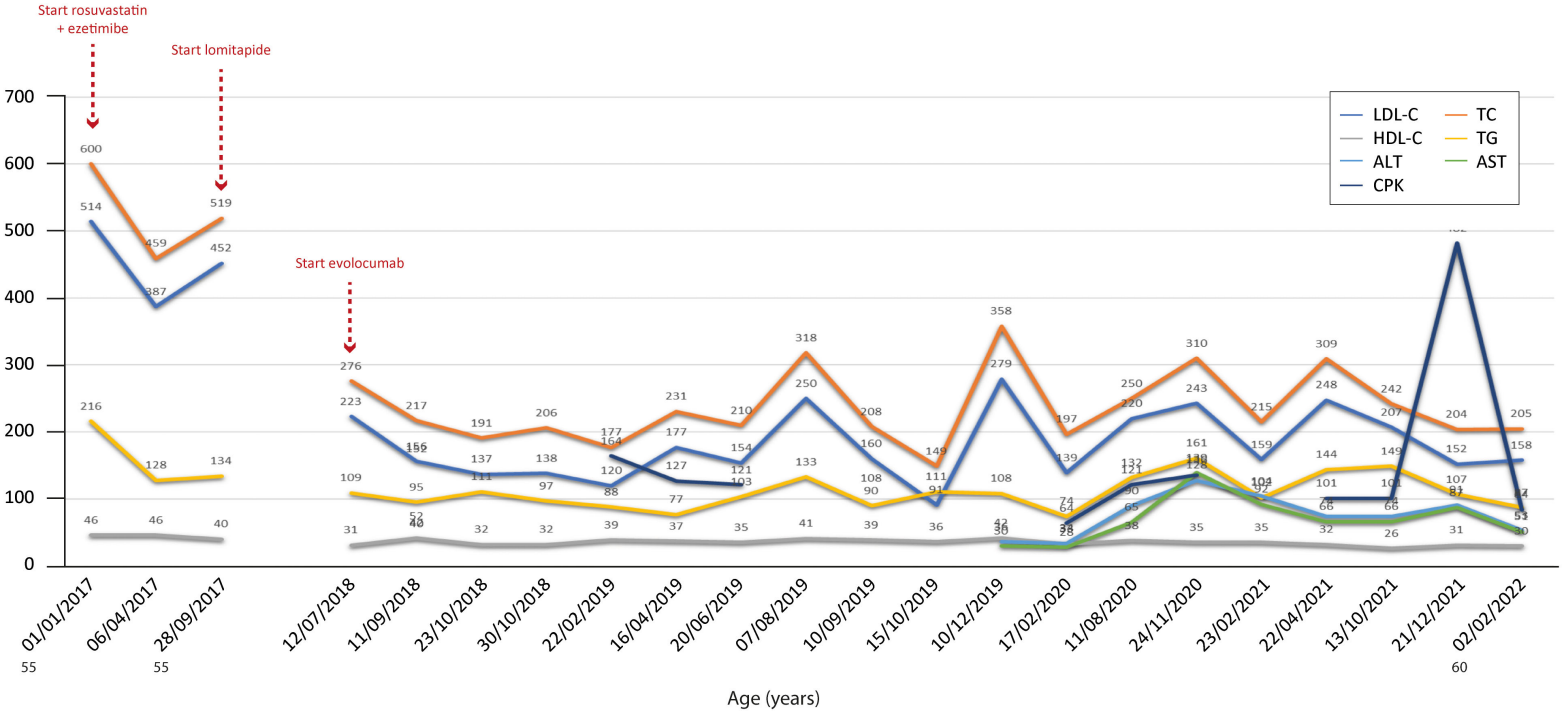
Temporal trends of cholesterol, hepatic enzymes and triglycerides in patient 3. The follow-up period was 5 years. Total and LDL cholesterol decreased with therapy relative to baseline values but fluctuated importantly over time, suggesting either incomplete therapy efficacy in this patient or an external contribution due to an inadequately balanced dietary regimen. In addition, a peak in CPK value was recorded at the end of the follow-up period. The y-axis represents the blood levels of the various biochemical parameters examined. TC, LDL cholesterol, TGs and HDL cholesterol are measured in mg/dL, whereas ALT, AST and CPK are measured in U/L. TC, total cholesterol; LDL, low-density lipoprotein; HDL, high-density lipoprotein; TG, triglycerides; ALT, alanine aminotransferase; AST, aspartate aminotransferase; CPK, creatinine phosphokinase.

**Figure 5:**
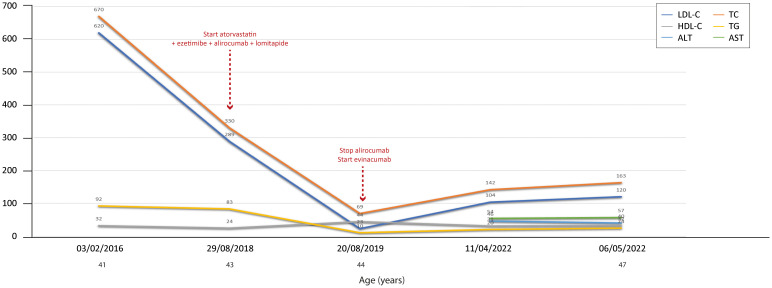
Temporal trends of cholesterol, hepatic enzyme, and triglycerides in patient 4. The follow-up period was 6 years. Compared with the other patients, the time point measurements of the various biochemical parameters were few for patient 4 despite the long follow-up period. A significant reduction in both total and LDL cholesterol occurred during therapy; only a slight increase in both parameters occurred by changing the PCSK9i used (alirocumab → evinacumab). The y-axis represents the blood levels of the various biochemical parameters examined. TC, LDL cholesterol, TGs and HDL cholesterol are measured in mg/dL, whereas ALT and AST are measured in U/L. TC, total cholesterol; LDL, low-density lipoprotein; HDL, high-density lipoprotein; TG, triglycerides; ALT, alanine aminotransferase; AST, aspartate aminotransferase.

**Figure 6:**
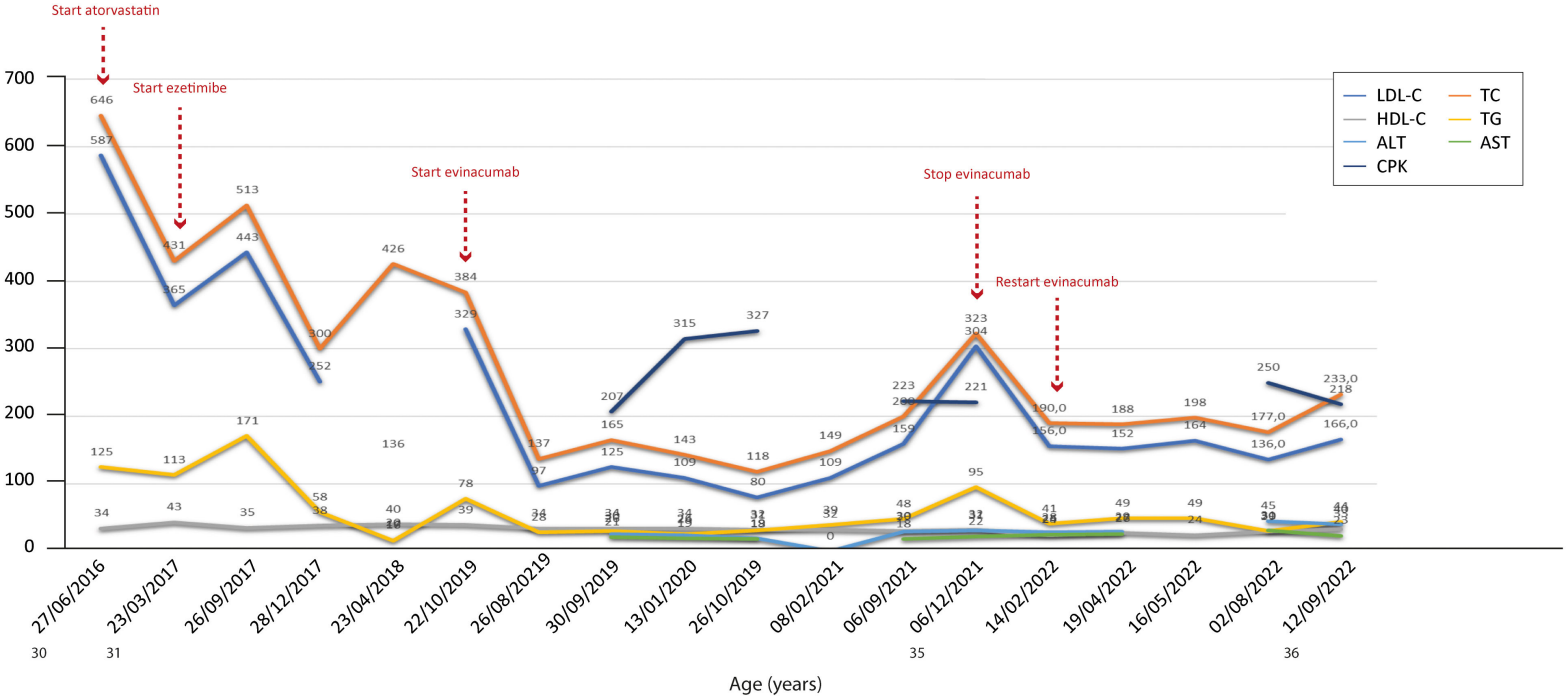
Temporal trends of cholesterol, hepatic enzymes and triglycerides in patient 5. The follow-up period was 6 years. Overall, the combined therapies induced a reduction in total and LDL cholesterol, with levels of both parameters increasing only after stopping evinacumab. The y-axis represents the blood levels of the various biochemical parameters examined. TC, LDL cholesterol, TGs and HDL cholesterol are measured in mg/dL, whereas ALT, AST and CPK are measured in U/L. TC, total cholesterol; LDL, low-density lipoprotein; HDL, high-density lipoprotein; TG, triglycerides; ALT, alanine aminotransferase; AST, aspartate aminotransferase; CPK, creatinine phosphokinase.

**Figure 7:**
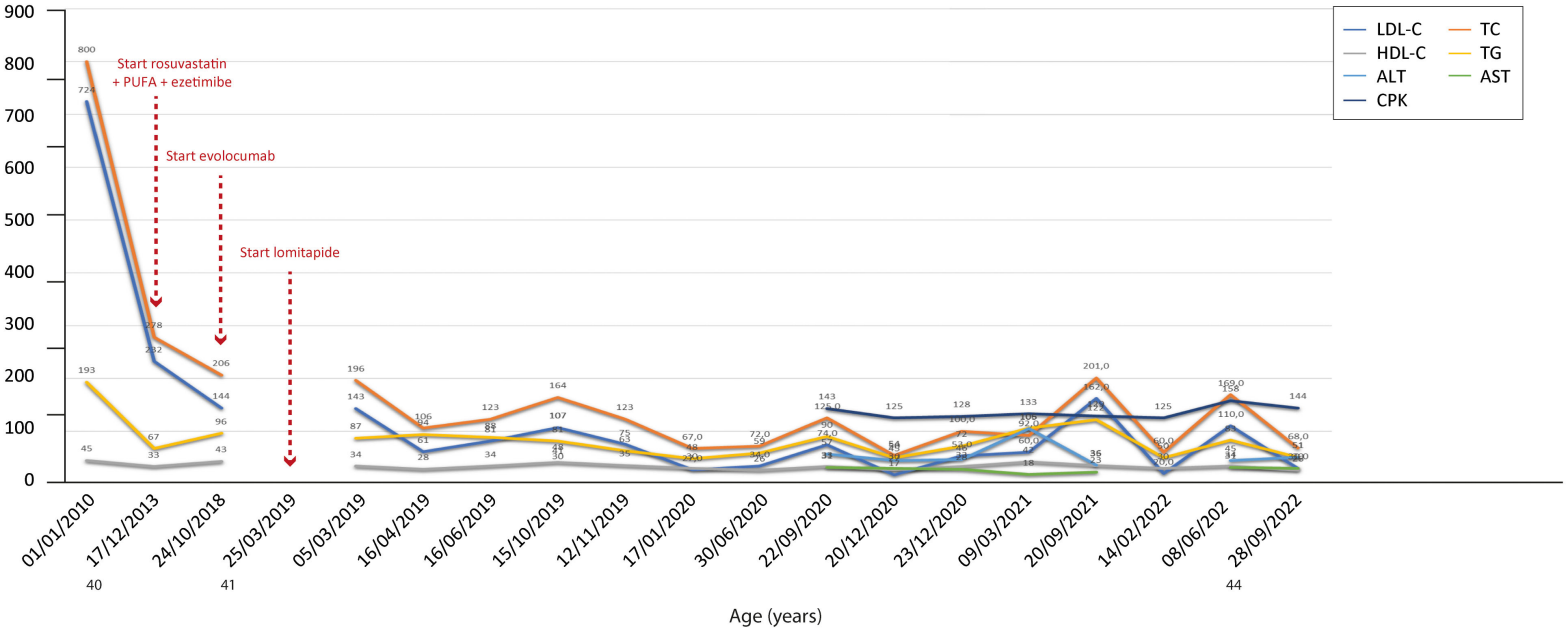
Temporal trends of cholesterol, hepatic enzymes and triglycerides in patient 6. The follow-up period was 12 years. In this patient, the standard LLT + novel pharmacological agents proved effective in reducing both total and LDL cholesterol levels. The y-axis represents the blood levels of the various biochemical parameters examined. TC, LDL cholesterol, TGs and HDL cholesterol are measured in mg/dL, whereas ALT, AST and CPK are measured in U/L. TC, total cholesterol; LDL, low-density lipoprotein; HDL, high-density lipoprotein; TG, triglycerides; ALT, alanine aminotransferase; AST, aspartate aminotransferase; CPK, creatinine phosphokinase.

**Figures 2**–**7** show the temporal trends during the follow-up period for the six patients concerning the various biochemical parameters examined above (some data are missing).

## 4 Conclusion

HoFH is a rare disease characterized by the possible risk of CV complications that can seriously threaten patients’ lives.

HoFH patients, due to their family and personal experience, are rigorous in the treatment and prevention of CV complications related to the disease itself. Until a few decades ago, the disease was burdened by early mortality due to CV events, as in the case of two patients (the older ones) described in this article who experienced sibling death in their early 20s. The lack of effective therapy to reduce LDL cholesterol, with the exception of apheresis, made the lives of these patients even more difficult. This led to frailty on the one hand and paranoia on the other hand against any symptoms, pushing them toward a sort of malnutrition in the belief that a very strict diet could somehow produce positive effects. A healthy diet is recommended to patients with HoFH to treat CV risk factors beyond cholesterol levels, as the final aim is prevention of CV events, while control of lipidemia is only achieved by pharmacologic treatments (4).

Fortunately, drug research and development have allowed to date for a robust treatment algorithm based on classical LLT plus the use of novel molecules, such as lomitapide; indeed, these pharmacological therapies provide better clinical management of patients (as also shown in our case series) and improve their quality of life. These therapies, particularly lomitapide, require careful monitoring because of the possible occurrence of side effects. Therefore, the proper use of diagnostic tools to monitor the health status of the body and appropriate management systems to deal with possible therapy-related complications are of paramount importance in the routine clinical practice of HoFH.

## Data Availability

The original contributions presented in the study are included in the article/**Supplementary Material**. Further inquiries can be directed to the corresponding author.
