# Supplementary material for: New insights into the management of homozygous familial hypercholesterolemia patients treated with lomitapide: a single-center experience
Source: Front Endocrinol (Lausanne). 2024 Dec 24;15:1515846. doi: 10.3389/fendo.2024.1515846 (PMC11703714; doi:10.3389/fendo.2024.1515846)
Supplement: Supplementary file 1 [file Table1.docx]

**Supplementary Table 1**. **Interaction between lomitapide and other drugs.**

| Drug | Activity on CYP450 or Pgp | Pharmacodynamic consequence | Possible clinical effect |
| --- | --- | --- | --- |
| CARDIOVASCULAR SECTOR | | | |
| Anti-platelet agents | | | |
| Clopidogrel | Lomitapide inhibits CYP450 | Increase in exposure to clopidogrel | Possible decrease in bioactivation of clopidogrel |
| Anticoagulants | | | |
| Heparin, enoxaparin, nadroparin, dalteparin | – | – | – |
| Warfarin, phenprocoumon, acenocoumarol | Lomitapide inhibits CYP450 | Possible increase in exposure to warfarin, phenprocoumon, and acenocoumarol | Possible increase in anticoagulant effect of warfarin, phenprocoumon, and acenocoumarol |
| GASTROINTESTINAL AND METABOLISM SECTOR | | | |
| Proton pump inhibitors | | | |
| Omeprazole, esomeprazole, pantoprazole | – | – | – |
| H2 antagonists | | | |
| Cimetidine | Cimetidine inhibits CYP450 | Increase in exposure to lomitapide | Possible increase in adverse effects of lomitapide |
| Ranitidine | – | – | – |
| HORMONES SECTOR | | | |
| Corticosteroids | | | |
| Prednisone, betamethasone | – | – | – |
| Dexamethasone | Dexamethasone induces CYP450 | Decrease in exposure to lomitapide | Possible decrease in lomitapide efficacy |
| Thyroid therapy | | | |
| Carbimazole | – | – | – |
| Levothyroxine | Levothyroxine inhibits CYP450 | Increase in exposure to lomitapide | Possible increase in adverse effects of lomitapide |
| Antineoplastic drugs | | | |
| Cyclophosphamide | Lomitapide inhibits CYP450 | Increase in exposure to cyclophosphamide | Possible decrease in bioactivation of cyclophosphamide |
| Antiestrogens | | | |
| Tamoxifen | Lomitapide inhibits CYP450 | Increase in exposure to tamoxifen | Possible increase in adverse effects of tamoxifen |
| ANTIMICROBIAL SECTOR | | | |
| Penicillins | | | |
| Amoxicillin, flucloxacillin | – | – | – |
| Cephalosporins | | | |
| Cefuroxime, cefpodoxime, ceftriaxone | – | – | – |
| Tetracyclines | | | |
| Doxycycline | – | – | – |
| Macrolides | | | |
| Clarithromycin | Clarithromycin inhibits CYP450 | Increase in exposure to lomitapide | Possible increase in adverse effects of lomitapide |
| Azithromycin | – | – | – |
| Erythromycin | Erythromycin inhibits CYP450 | Increase in exposure to lomitapide | Possible increase in adverse effects of lomitapide |
| Quinolones | | | |
| Ciprofloxacin | Ciprofloxacin increases the period of QT; lomitapide does not increase the QTc interval (no additive effect). Consider whether to monitor ECG on an individual basis. | – | – |
| Levofloxacin, norfloxacin | Levofloxacin and norfloxacin increase the period of QT; lomitapide does not increase the QTc interval (no additive effect). Consider whether to monitor ECG on an individual basis. | – | – |
| Sulfonamides | | | |
| Co-trimoxazole | Lomitapide inhibits CYP450 | Increase in exposure to co-trimoxazole | Possible increase in adverse effects of co-trimoxazole |
| Azoles | | | |
| Itraconazole, fluconazole, voriconazole, ketoconazole | Itraconazole, fluconazole, voriconazole, and ketoconazole inhibit CYP450 and increase the period of QT; lomitapide does not increase the QTc interval (no additive effect). Consider whether to monitor ECG on individual basis | Increase in exposure to lomitapide | Possible increase in adverse effects of lomitapide |
| Allylamine | | | |
| Terbinafine | Lomitapide inhibits CYP450 | Increase in exposure to terbinafine | Possible increase in adverse effects of terbinafine |
| Nitroimidazoles | | | |
| Metronidazole | – | – | – |
| Antivirals/nucleoside analogues | | | |
| Acyclovir, valacyclovir, ganciclovir, valganciclovir | – | – | – |
| Antimycobacterials | | | |
| Rifampicin | Rifampicin induces CYP450 | Decrease in exposure to lomitapide | Possible decrease in Lomitapide efficacy |
| Isoniazid, ethambutol | – | – | – |
| Protease inhibitors | | | |
| Ritonavir | Ritonavir inhibits CYP450 and increases the period of QT; lomitapide does not increase the QTc interval (no additive effect). Consider whether to monitor ECG on an individual basis | Increase in exposure to lomitapide | Possible increase in adverse effects of lomitapide |
| Saquinavir | Lomitapide inhibits CYP450 and Pgp | Increase in exposure to saquinavir | Possible increase in adverse effects of saquinavir |
| Darunavir | Darunavir inhibits CYP450 | Increase in exposure to lomitapide | Possible increase in adverse effects of Lomitapide |
| Atazanavir | Lomitapide inhibits CYP450 and Pgp | Increase in exposure to atazanavir | Possible increase in adverse effects of atazanavir |
| Lopinavir | Lomitapide inhibits CYP450 and Pgp; lopinavir inhibits CYP450 | Increase in exposure to lopinavir, lopinavir increases exposure to lomitapide | Possible increase in adverse effects of lopinavir and lomitapide |
| Indinavir | Lomitapide inhibits CYP450 and Pgp | Increase in exposure to indinavir | Possible increase in adverse effects of indinavir |
| Nucleoside and nucleotide reverse transcriptase inhibitors | | | |
| Lamivudine, emtricitabine, zidovudine | – | – | – |
| Non-nucleoside reverse transcriptase inhibitors | | | |
| Efavirenz | Lomitapide inhibits CYP450, efavirenz induces CYP450 | Increase in exposure to efavirenz and decrease in exposure to lomitapide | Possible increase in adverse effects of efavirenz, possible decrease in lomitapide efficacy |
| Nevirapine | Lomitapide inhibits CYP450, nevirapine induces CYP450 | Increase in exposure to nevirapine and decrease in exposure to lomitapide | Possible increase in adverse effects of nevirapine, possible decrease in lomitapide efficacy |
| Etravirine | Lomitapide inhibits CYP450, etravirine induces CYP450 | Increase in exposure to etravirine and decrease in exposure to lomitapide | Possible increase in adverse effects of etravirine, a possible decrease in lomitapide efficacy |
| Antimalarials | | | |
| Proguanil, atovaquone, doxycycline | – | – | – |
| Quinine, mefloquine | Lomitapide inhibits CYP450, quinine and mefloquine increase the period of QT; lomitapide does not increase the QTc interval (no additive effect). Consider whether to monitor ECG on an individual basis | Increase in exposure to quinine and mefloquine | Possible increase in adverse effects of quinine and mefloquine |
| Chloroquine | Chloroquine increases the period of QT; lomitapide does not increase the QTc interval (no additive effect). Consider whether to monitor ECG on an individual basis. This implies monitoring ECG | – | – |
| IMMUNOMODULATORS SECTOR | | | |
| Immunosuppressors | | | |
| Tacrolimus, sirolimus, everolimus | Lomitapide inhibits CYP450 | Increase in exposure to tacrolimus, sirolimus, everolimus, and cyclosporine | Possible increase in adverse effects of tacrolimus, sirolimus, everolimus, and cyclosporine |
| Cyclosporine | Cyclosporine inhibits CYP450 | Increase in exposure to lomitapide | Possible increase in adverse effects of lomitapide |
| Mycophenolate mofetil, methotrexate, azathioprine | – | – | – |
| MUSCULOSKELETAL SECTOR | | | |
| Non-steroidal anti-inflammatory drugs | | | |
| Metamizole (i.e., Novalgine) | Metamizole induces CYP450 | Decrease in exposure to lomitapide | Possible decrease in lomitapide efficacy |
| Acetylsalicylic acid | - | - | - |
| Ibuprofen, mefenamic acid, diclofenac | These drugs are metabolized by some subforms of CYP450 that do not appear to be inhibited by lomitapide | Caution! Being Lomitapide a CYP450 inhibitor, a possible increase in exposure to ibuprofen, mefenamic acid, and diclofenac is possible. | Caution! Possible increase in adverse effects of ibuprofen, mefenamic acid, and diclofenac. |
| Anti-gout drugs | | | |
| Allopurinol | – | – | – |
| NERVOUS SYSTEM SECTOR | | | |
| Opioids | | | |
| Tramadol, methadone, oxycodone, buprenorphine | Lomitapide inhibits CYP450 | Increase in exposure to tramadol, methadone, oxycodone, and buprenorphine | Possible increase in adverse effects of tramadol, methadone, oxycodone, and buprenorphine |
| Hydromorphone, morphine | – | – | – |
| Other therapies | | | |
| Paracetamol | – | – | – |
| Anti-migraine | | | |
| Dihydroergotamine | Lomitapide inhibits CYP450 | Increase in exposure to dihydroergotamine | Possible increase in adverse effects of dihydroergotamine |
| Sumatriptan | – | – | – |
| RESPIRATORY SYSTEM SECTOR | | | |
| H1 antagonists | | | |
| Cetirizine, levocetirizine, loratadine, fexofenadine | Lomitapide inhibits Pgp | Increase in exposure to cetirizine, levocetirizine, loratadine, and fexofenadine | Possible increase in adverse effects of cetirizine, levocetirizine, loratadine, and fexofenadine |
| Anti-asthmatics | | | |
| Salbutamol, theophylline | – | – | – |
| OTHER POSSIBLE SUBSTANCES INTERACTING WITH LOMITAPIDE | | | |
| St. John's wort | St. John's wort induces CYP450 | Decrease in exposure to lomitapide | Possible decrease in lomitapide efficacy |
| Grapefruit | Grapefruit induces CYP450 | Increase in exposure to lomitapide | Possible increase in adverse effects of lomitapide |
| Liquorice | Liquorice induces CYP450 | Increase in exposure to lomitapide | Possible increase in adverse effects of lomitapide |
| *CYP450: cytochrome P-450; Pgp: permeability glycoprotein, also known as multidrug resistance protein 1 (MDR1).* | | | |
